# Fibroblast Growth Factor 13 Facilitates Peripheral Nerve Regeneration through Maintaining Microtubule Stability

**DOI:** 10.1155/2021/5481228

**Published:** 2021-08-20

**Authors:** Rui Li, Xuetao Tao, Minghong Huang, Yan Peng, Jiahong Liang, Yanqing Wu, Yongsheng Jiang

**Affiliations:** ^1^The Affiliated Xiangshan Hospital of Wenzhou Medial University, No. 291 Donggu Road, Xiangshan County, Zhejiang 315000, China; ^2^PCFM Lab, GD HPPC Lab, School of Chemistry, Sun Yat-sen University, Guangzhou 510275, China; ^3^The Second Affiliated Hospital, Zhejiang University School of Medicine, Hangzhou, Zhejiang 310009, China; ^4^Hangzhou Institute for Food and Drug Control, Hangzhou, Zhejiang 310014, China; ^5^Betta Pharmaceuticals Co., Ltd, Hangzhou, Zhejiang 310000, China; ^6^The Institute of Life Sciences, Engineering Laboratory of Zhejiang Province for Pharmaceutical Development of Growth Factors, Biomedical Collaborative Innovation Center of Wenzhou, Wenzhou University, Wenzhou, Zhejiang 325035, China

## Abstract

Peripheral nerve injury (PNI), resulting in the impairment of myelin sheaths and axons, seriously affects the transmission of sensory or motor nerves. Growth factors (GFs) provide a biological microenvironment for supporting nerve regrowth and have become a promising alternative for repairing PNI. As one number of intracellular growth factor family, fibroblast growth factor 13 (FGF13) was regard as a microtubule-stabilizing protein for regulating cytoskeletal plasticity and neuronal polarization. However, the therapeutic efficiency and underlying mechanism of FGF13 for treating PNI remained unknown. Here, the application of lentivirus that overexpressed FGF13 was delivered directly to the lesion site of transverse sciatic nerve for promoting peripheral nerve regeneration. Through behavioral analysis and histological and ultrastructure examinations, we found that FGF13 not only facilitated motor and sense functional recovery but also enhanced axon elongation and remyelination. Furthermore, pretreatment with FGF13 also promoted Schwann cell (SC) viability and upregulated the expression cellular microtubule-associated proteins *in vitro* PNI model. These data indicated FGF13 therapeutic effect was closely related to maintain cellular microtubule stability. Thus, this work provides the evident that FGF13-medicated microtubule stability is necessary for promoting peripheral nerve repair following PNI, highlighting the potential therapeutic value of FGF13 on ameliorating injured nerve recovery.

## 1. Introduction

Peripheral nerve injury (PNI) is one of the most traumatic disorders for triggering a decrease or a complete loss of motor and sensory function in clinical practice [1]. It has been reported that an estimated annual incidence of 67,800 major PNIs occur in the United States alone, and the annual health-care dollars per individual were approximately $150 billion, causing an enormous socioeconomic burden [2, 3]. Despite the developing peripheral nerve has an intrinsic capability for regeneration, the outcomes of optimal structural and functional recovery are not always satisfactory [4]. Recent advances in nerve reconstruction and autologous nerve grafting remain the gold standard technique for the repair of acute nerve injury; however, these treatments exist some limitations, including the limited amount of autologous donor nerves, neuronal mismatch between the donor and the recipient site, even neuroma formation at the donor site [5, 6]. To overcome these limitations, current therapeutic strategies are focused on administration of exogenous growth factors (GFs) as a therapeutic relevant agent to ameliorate axonal regrowth and remyelination following PNI [7].

GFs refer to a neurotrophic factor family of bioactive cytokines with the ability to regulate cellular proliferation, migration, and differentiation. Several preclinical trials have described that exogenous application of GFs to the lesion site of peripheral nerve is able to stimulate axonal sprouting, myelination, neurogenesis, and/or neovascularization [8–10]. These encouraging outcomes reveal that GFs may act as the potent therapeutic drugs for repairing PNI. As an intracrine protein of the GF subfamily, fibroblast growth factor 13 (FGF 13) is not only widely distributed in human adult and developing brain but also overexpressed in the mammalian heart. Initially, FGF13 is regarded as a candidate gene for diagnosing X-chromosome-linked mental retardation (XLMR) and negatively enhancing caveolae-mediated mechanoprotection [11, 12]. In the subsequent evaluation, FGF13 has been identified as a microtubule-stabilizing agent for guiding growth cone initiation, neuronal polarization, and axonal extension. For instance, lentivirus-mediated FGF13 overexpression could increase the level of microtubule stabilizing proteins to enhance axon regeneration and functional recovery after spinal cord injury (SCI) [13]. In early embryonic development, FGF13 also participated in neural differentiation via MEK5-ERK5 pathway [14]. Additionally, FGF13-deficient mice exhibited learning and memory impairment due to the imbalance of microtubule assembly in neocortex and hippocampus region [15]. Based on the above fact, we try to reveal whether FGF13 also plays an essential role on restoring PNI recovery.

As one component of cytoskeleton, microtubule provides an organizational framework to control neuronal extension, retraction, and steering through modifying growth cone dynamic growth and shrinkage. In living cells, microtubule stabilization is critical for neural polarization and axon formation, which can be reflected by detecting the content of microtubule-associated proteins (MAPs), such as acetylated- (Ace-) tubulin, tyrosinated- (Tyr-) tubulin, Tau, Kinesin-5, and Dynein. Increasingly, evidences have suggested that microtubule stability was closely associated with neuronal traumatic diseases [16]. Manipulation of microtubule stabilization with Taxol could reduce fibrotic scar formation and enable axonal regeneration after SCI via facilitating intrinsic axon growth capacity [17]. In line with this efficacy, systemic administration of epothilone B further induced microtubule polymerization into the neurite tips to restore axon elongation under an inhibitory environment after SCI [18]. Mechanistically, this positive feedback was modulated by a specific autophagy-inducing peptide, Tat-beclin1, suggesting a critical role of autophagy in maintaining microtubule stability [19]. Given that axon regeneration and scarring reduction appear to rely indirectly or directly on microtubule dynamics and stability, this mechanism may possibly play an instructive role in axon formation and remyelination following PNI.

In this work, we aim to identify whether FGF13 has a certain capability for improving the injured peripheral nerve regeneration in a rat model and reveal its underlying molecular mechanism via various comprehensive evaluations, including histological, morphological, and functional assessments. Our data proved that overexpression of FGF13 could maintain microtubule stabilization in Schwann cells (SCs) to promote their survival, axon regrowth, and remyelination, resulting in the improvement of locomotor recovery following PNI. These results suggest, for the first time, that supplement of FGF13 is an effective and feasible therapeutic strategy for rehabilitating PNI.

## 2. Materials and Methods

### 2.1. Animals and Ethics Statement

Forty male Sprague-Dawley rats, weighing from 200 to 220 g, were obtained from the Animal Center of the Chinese Academy of Science (Shanghai, China). The rats were individually housed in wire bottom cages under humidity (50-60%) and temperature (23-25°C) controlled conditions with a 12 h light/dark cycle. The animals had free access to water and food. All protocols involving animal care and experimental procedures were reviewed and approved by the Chinese National Institutes of Health.

### 2.2. Preparation of Nerve Transection Model and Drug Application

For the surgical procedure of sciatic nerve transection model, after anesthetizing with 4% pentobarbital sodium (30 mg/kg, i.p. injection), the rats were fixed on the operating table and shaved the hair on the right thigh. Then, the right sciatic nerve was isolated and exposed carefully by cutting skin and blunt splitting dorsolateral gluteal muscles. Next, the sciatic nerve was transected at approximately 0.5 cm distal to the sciatic notch using fresh scalpel blade. The proximal and distal ends of the cut nerves were sutured by 10/0 polypropylene sutures (Ethicon, Somerville, NY) under a microscope, followed by suturing the muscles and skin using 7/0 sutures.

After surgery, all the tested rats were randomly divided into three groups: a FGF13 lentiviral vector-treated group (FGF13), a blank lentiviral vector-treated group (vehicle), and a saline-treated group (PNI). Each group was consisted of 10 rats. For the FGF13 group, each animal was orthotopical injection of 10 *μ*l (2 × 10^8^ TU ml^−1^) [13] lentivirus (LV) which expressed FGF13 into the sutured site via a Hamilton microsyringe at a rate of 500 nl min^−1^. The LV-FGF13 were purchased from Shanghai GeneChem Co., Ltd. (Shanghai, China). The primer of lentiviral particles of FGF13 sequence was used forward: 5′-CCAACTTTGTGCCAACCGGTCGCCACCATGGCTTTGTTAAGGAAGTC-3′, reverse: 5′-AATGCCAACTCTGAGCTTCGTTGATTCATTGTGGCTCATG-3′. The vehicle group was administrated the same dosages of lentiviral vector. After injection, the needle was left in place for an additional 10 min and then slowly withdraw. Similarly, the rats in the PNI group received the same dose of saline. For the sham operation (control) group, the animals underwent only the surgical procedure without affecting the sciatic nerve.

### 2.3. Functional Recovery Analysis

The neurological recovery in all testing animals was evaluated through walking track analysis and von Frey hair test once a week for 6 weeks after surgery. The walking track analysis was quantified using the sciatic function index (SFI), which was calculated as the following formula proposed by de Medinaceli et al. [20]: SFI = (−38.3 × (EPL − NPL)/NPL) + (109.5 × (ETS − NTS)/NTS) + (13.3 × (EIT − NIT)/NIT) − 8.8. These indexes were taken both from the foot of the experimental foot print length (EPL), experimental foot toe spread (ETS), experimental foot intermediary toe (EIT), and nonoperated side (NPL, NTS, and NIT). The SFI value of about 0 represents normal recovery, while the SFI value close to -100 indicates total impairment.

The mechanical allodynia threshold was evaluated by von Frey hair test which described elsewhere [21]. Briefly, all the experimental rats were habituated in a glass cubicle on an elevated wire mesh platform for at least 1 hour. Then, the right hind paw in each animal was stimulated with a series of calibrated monofilaments (Stoelting, Wood Dale, IL). Monofilaments were applied with a constant increasing pressure until a perceptible bend of approximately 90° for lasting 2 seconds. At this moment, the monofilament of definitive ascending force was also recorded as the positive withdrawal force. The experimental process was repeated three times with an interval of 15 minutes by the same investigator who was blinded to the group design.

### 2.4. Tissue Preparation

At postoperative 6 weeks, all rats were anesthetized and sacrificed to harvest the regenerated sciatic nerves and gastrocnemius muscle. Meanwhile, the wet muscle weight in each rat was recorded. For histological staining, the nerve and muscle tissues were fixed with 4% paraformaldehyde at 4°C for 24 h. Subsequently, the samples were embedded in paraffin, cut into longitudinal or transverse sections of 5 *μ*m thickness using a microtome (Thermo Fisher Scientific HM 315, Waltham, USA), and mounted on slides. For immunofluorescence staining, the 0.5 cm segment of regenerative nerve was embedded in optimal cutting temperature compound (OCT, Sakura Tokyo, Japan) and cut into 10 *μ*m sections using a cryostat (Leica Microsystems Wetzlar GmbH, Hesse-Darmstadt, Germany). For immunoblotting and RT-PCR detection, the harvested sciatic nerve segment (0.5 cm length) taken from the lesion regions was immediately stored at -80°C.

### 2.5. Histological Staining and Morphometric Analysis

The serial paraffin sections were deparaffinized and rehydrated with xylene and a gradient ethanol solution. Thereafter, both of nerve and muscle sections were received Hematoxylin and Eosin (H&E) staining using H&E kit (Beyotime Institute of Biotechnology, China). For muscle sections, they were stained with H&E hematoxylin and eosin reagents for 5 min and 1 min, respectively. For nerve tissue sections, they were stained with these two dyes for 5 min and 8 min, respectively. The nerve sections also performed Masson's trichrome staining using Masson's trichrome staining kit (Solarbio, G1340) according to the instructions. Briefly, sections were first underwent deparaffinage and rehydration as describe by H&E staining, followed by staining with nucleus, fibrous, and collagen using hematoxylin, ponceau acid fuchsine solution, and aniline blue for 5 min, 5 min, and 20 s, respectively. After dehydrating, slides were mounted with neutral resin and covered by cove using rslips. In the end, all staining sections were observed and photographed using a Nikon microscope (Nikon, Tokyo, Japan).

The myelin sheath regeneration was also evaluated by transmission electromicroscope observation as previously described elsewhere [22]. Briefly, 2 mm regenerated nerve samples were fixed with 2.5% glutaraldehyde for 4 h, followed by postfixing with 1% osmium and 1% uranyl acetate for 1 h, respectively. After dehydrating in a graded acetone series, the samples were embedded in an Epon 812 resin and cut into semithin sections (thickness: 1.0 *μ*m) for toluidine blue staining. The ultrathin cross sections with a thickness of 50–60 nm were also prepared and then were stained with lead citrate and uranyl acetate for the transmission electron microscope observation (TEM; HT7700, Hitachi, Ltd., Japan).

**Figure 1:**
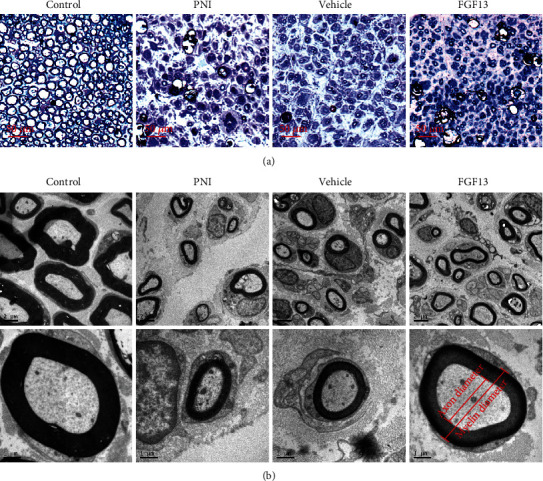
Analysis of regenerating cables at week 6 after injury. (a) Representative semithin transverse sections of regenerated nerves stained by toluidine blue in four groups; (b) TEM of ultrathin sectioned nerves of the four groups. The gross myelin distribution and regeneration were clearly seen via the lower magnification (2 *μ*m) in the middle panel. The bottom panel enlarged images with the higher magnification of 1 *μ*m were representative individual myelin in each group and not from the middle panel.

For the myelinated indexes including myelin counts, diameter of myelin sheath, thickness of myelin sheath, and G-ratio were calculated using the following two equations:

(1) $$\begin{array}{c} \text{Thickness of myelin sheath}=\frac{\left(\text{Myelin diameter}-\text{Axon diameter}\right)}{2}, \end{array}$$

(2) $$\begin{array}{c} \text{G}-\text{ratio}=\frac{\text{Axon diameter}}{\text{Myelin diameter}}, \end{array}$$

where for individual myelin, the way for measuring axon diameter and myelin diameter per myelin was according to the bottom panel enlarged images in Figure 1(b). Quantification of myelin counts, axon diameter, and myelin diameter could be automatically obtained using the ImageJ software. The data of myelin counts were performed through measuring five randomly selected fields in each animal. For quantifying axon and myelin diameter, 70 myelin sheaths were randomly selected from at least 10 images per animal. Each group contained 5 animals. All counting were quantified by two blinded independent observers who were blind to the experimental procedures and samples.

### 2.6. Immunofluorescence

After blocking with 5% BSA for 30 min, frozen sections were colabeled with anti-NF-200 (1 : 100,000, ab4680, Abcam) and anti-MBP (1 : 1000, ab40390, Abcam) primary antibodies, overnight at 4°C. Then, FITC-conjugated anti-rabbit IgG (Abcam, ab150073) and TRITC-conjugated anti-mouse IgG (Abcam, ab7065) were diluted with PBS at the concentration of 0.67 *μ*g/ml and dropped in the tissue samples, followed by incubating at 37°C for 1 h in dark condition. After staining nuclei with 0.1% of 4,6-diamidino-2-phenylindole (DAPI) for 5 min, the stained sections were observed and imaged using a confocal fluorescence microscope (Nikon, Japan).

**Figure 2:**
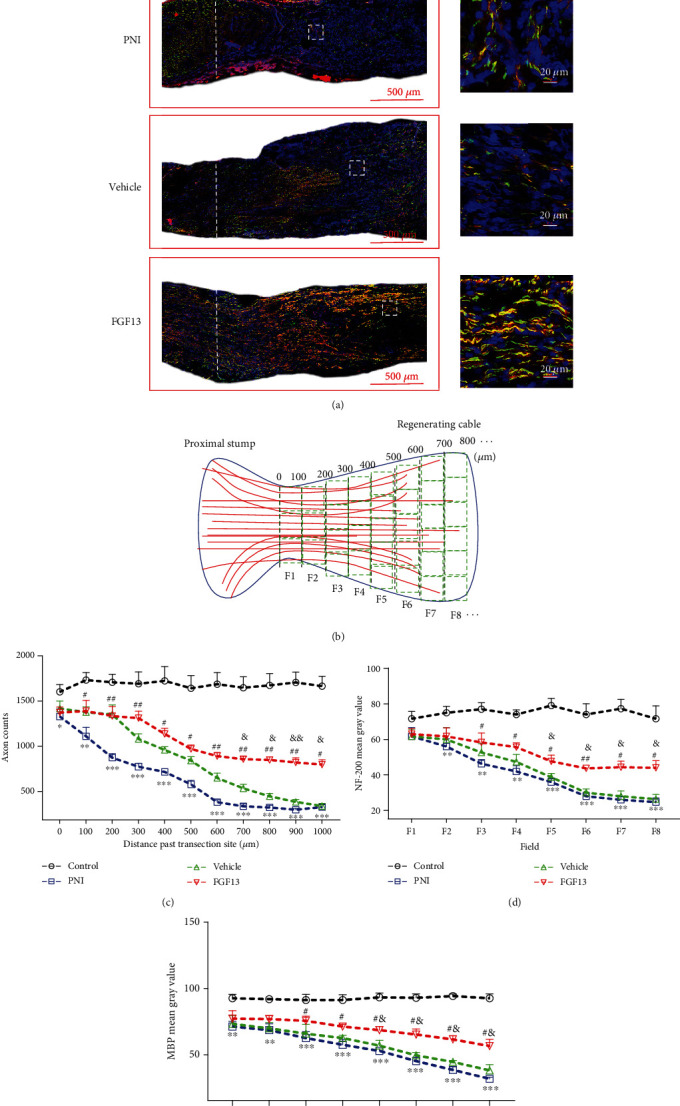
Histological analyses of the regenerated sciatic nerves in each group at 6 weeks after transection injury. (a) Representative costaining images for NF-200 and MBP of longitudinal sections in each experimental group. The scale bars in lower and higher magnifications were 500 *μ*m and 20 *μ*m, respectively. (b) Schema of nerve regeneration counting method proximal to the original transection through fields of the corresponding regenerating cable. (c) Quantification of total axon numbers in different fields of the indicated groups. (d, e) Quantitative analyses of average fluorescence intensity for NF-200 and MBP in indicated region of whole regenerated cable. Data are shown as means ± SEM, *n* = 5. PNI vs. control: ^∗^*P* < 0.05, ^∗∗^*P* < 0.01, and ^∗∗∗^*P* < 0.001; FGF13 vs. vehicle: ^&^*P* < 0.05 and ^&&^*P* < 0.01; FGF13 vs. PNI: ^#^*P* < 0.05 and ^##^*P* < 0.01.

With the observer blinded to the identity of the groups, approximately three-fourths sections of the regenerating bridge were photographed at high-power light microscopy (400x). Photographs were divided into several regions with 100 *μ*m interval. Axon numbers, NF200^+^ and MBP^+^ positive signals in each individual region of longitudinal section of the nerve cable, were analyzed using the ImageJ software as previously described [23]. The diagrammatic sketch of regional dividualing and identifying was shown in Figure 2(b). Three sections per animal and three animals per group were randomly selected for statistical analysis.

### 2.7. Immunoblotting

Total protein was extracted from regenerated sciatic nerve using RIPA lysis buffer containing 1% protease and phosphatase inhibitors. The protein concentration in different samples was measured using the BCA Assay Kit. A total of 80 *μ*g of protein was separated by 12% (*w*/*v*) gels and then transferred to a PVDF membrane (Millipore), followed by blocking with 5% *w*/*v* nonfat milk for 1.5 h. After washing with TBST for three times, the PVDF membranes were incubated overnight at 4°C with primary antibodies targeting the following proteins: Ace-tubulin (1 : 1000, CST, Catalog No. #5335), Tyr-tubulin (1 : 500, Sigma, Catalog No. T9026), Tau (1 : 1000, Abcam, Catalog No. ab92676), Kinesin-5 (1 : 1000, Abcam, Catalog No. ab167429), Dynein (1 : 1000, Abcam, Catalog No. ab171964), Bax (1 : 500, CST, Catalog No. #14796), Bcl-2 (1 : 500, CST, Catalog No. #4223), and Cleaved caspase-3 (1 : 500, Abcam, Catalog No. ab2302). The next day, blots were washed in TBST and incubated with HRP-labeled secondary antibodies (1 : 2000 dilution) for 1 h at 25°C temperature. Immunobands were visualized by ChemiDocXRS + Imaging System, and band intensity was quantified by the ImageJ software.

### 2.8. Preparation of Myelin Extracts

Myelin debris were extracted from uncut sciatic nerve in adult rats as described in previous with modification [8]. Briefly, the collecting sciatic nerves were homogenized in 0.32 M sucrose using a homogenizer (PRO 200, USA), followed by centrifuging twice at 1,5000 g for 1 h. After removal of sucrose, myelin fractions were added PBS and filtered through 0.22 *μ*m filters to sterilize and remove any particulate substance.

### 2.9. Cell Culture, Transfection, and Treatments

The RSC 96 cells (a rat Schwann cell line) were purchased from the cell bank of the Chinese Academy of Sciences (Shanghai, China). Before transfection, cells were maintained in Dulbecco's modified Eagle Medium (DMEM, Gibco) containing 10% foetal bovine serum (FBS, Thermo Fisher Scientific) and incubated in a humidified atmosphere containing 5% CO_2_ at 37°C. At three passages, the cells were seeded on 6-well culture plates at an approximate density of 5 × 10^4^/cm^2^ and grown for 24 h to approximately 70% confluency. Afterwards, the old medium were replaced with fresh serum-free medium; meanwhile, the cells were infected with recombinant lentiviral construct to overexpression FGF13 with the concentration of 1 × 10^8^ TU/ml or empty lentiviral vectors (LV-vector) with the same concentration. After 24 h incubation, the cells were supplemented with normal growth medium containing 10 *μ*g/ml myelin extracts for culturing another 2 days. The lentivirus expression vector (GV358) for overexpression FGF13 (LV-FGF13) was constructed by Genechem (Shanghai, China). We regarded the cell culturing in normal medium as the control group. The medium contains only myelin debris as the myelin group. The cells transfecting LV-FGF13 and myelin added were taken as the myelin+FGF13 group.

### 2.10. Live/Dead Staining Assay

Apoptotic cell death was quantified using the Live/Dead Viability Assay kit (L3224, Thermo Fisher) according the manufacturer's instructions. Briefly, after gently washing in PBS 3 times, the medium culturing RSC 96 cells were added 1 *μ*M Calcein-AM and 2 *μ*M propidium iodide for incubating 30 min at room temperature in the dark condition. Afterwards, the live cells were observed using a 490 nm excitation filter, whereas the dead cells were observed using a 545 nm excitation filter under a laser scanning confocal microcopy system (LSCM, Nikon, Z2). The live and dead cells were automatically calculated using the ImageJ software. The cell viability rate in each group was calculated as following equation: The cell viability rate (%) = the live cell mumbers/total cell numbers × 100%. Five random fields were counted for each sample, and this experiment was performed in triplicate.

### 2.11. Statistical Analysis

All quantitative data are expressed as means ± SEM. Statistical comparisons were performed using the GraphPad Prism 7 Software (GraphPad Software Inc., La Jolla, CA, USA). For two-group comparison, statistical significance was analyzed by Student's *t*-test. For multiple group comparison, the statistical evaluation of the data was performed using one-way analysis of variance (ANOVA) followed by post hoc Tukey's test. In all the analyses, differences were considered significant at *P* < 0.05. Each experiment was performed at least three times to ensure accuracy.

## 3. Result

### 3.1. FGF13 Persistently Facilitates Neurologic Functional Recovery after PNI

**Figure 3:**
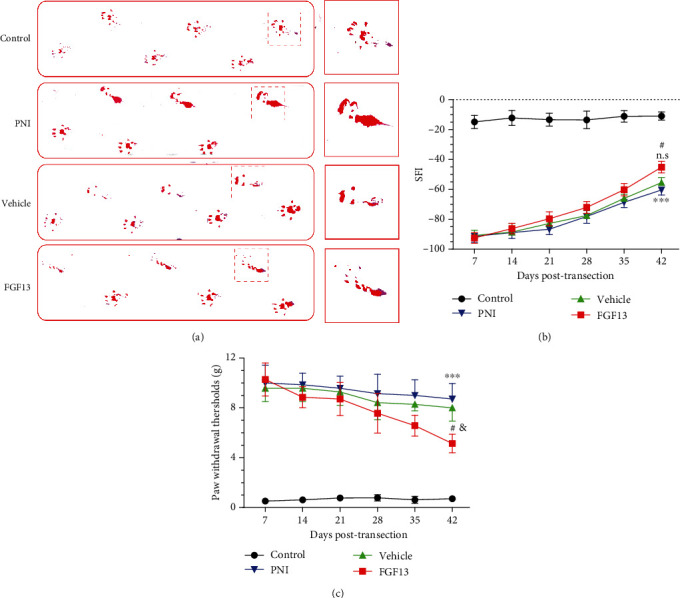
FGF13 promotes functional recovery after PNI: (a) representative images of the rats' footprints in the control, PNI, vehicle, and FGF13 groups at 6 weeks after treatment; (b) SFI analysis of the different groups at 1, 2, 3, 4, 5, and 6 weeks after operation; (c) withdrawal threshold was determined using von Frey hair test at indicated time points. Values are expressed as mean ± SEM, *n* = 5. PNI vs. sham: ^∗∗∗^*P* < 0.001; FGF13 vs. vehicle: ^&^*P* < 0.05, n.s representing not statistical significance; FGF13 vs. PNI: ^#^*P* < 0.05.

To evaluate the motor function recovery in all groups, walking track analysis was performed at determine time postsurgery. Representative foot prints at 6 week postoperation from each group were shown in Figure 3(a). The result showed that the operative footprints in the FGF13 treating group had superior toe spread when compared with the PNI and vehicle groups. Meanwhile, the SFI values in all surgery groups were gradually became increased as time elapsed. It was worth noting that the SFI values in the FGF13 group were significantly higher than those in the PNI and vehicle groups at 6 weeks after surgery (Figure 3(b), *P* < 0.05). Additionally, recovery of sensory function was monitored weekly using the von Frey hair test. Consistent with the motor function evaluation, overexpression of FGF13 significantly accelerated sensory recovery following transection injury. At 6 weeks postsurgery, the paw withdrawal thresholds in FGF13-treated rats were approximately twofold than the only saline-treated PNI rats (Figure 3(c)).

### 3.2. FGF13 Attenuates Atrophy of the Gastrocnemius Muscle after PNI

**Figure 4:**
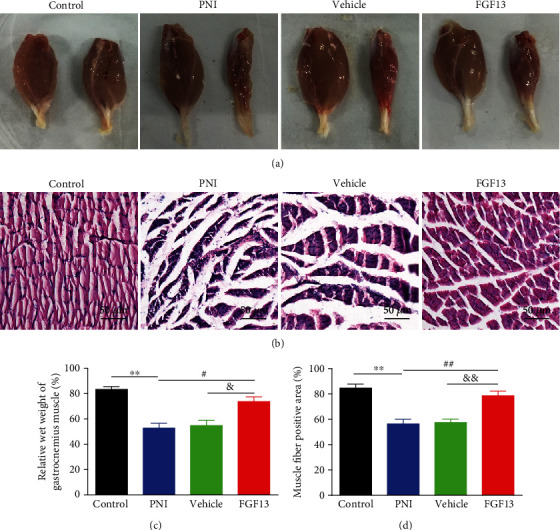
The morphological and microstructural analyses of gastrocnemius muscle. (a) The gross observation of the gastrocnemius muscles in the control, PNI, vehicle, and FGF13 groups at week 6 after the transection. (b) H&E staining of the cross-sections of the muscles in the four groups. Scale bar = 50 *μ*m. (c) Quantification of the relative muscle weight from (a). (d) Quantification of the positive area of muscle fibers in the indicated groups from (b). Values are expressed as mean ± SEM, *n* = 5. ^∗∗^*P* < 0.01 vs. the control group; ^&^*P* < 0.05 and ^&&^*P* < 0.01 vs. the PNI group; ^#^*P* < 0.05 and ^##^*P* < 0.01 vs. the PNI group.

Gastrocnemius muscle reinnervation, characterized by the increase of muscle weight and muscle fiber area, represents the functional recovery of the sciatic nerve [24]. We measured the isolated gastrocnemius muscle weights to evaluate the muscle atrophy and performed H&E staining to investigate the morphological changes in the gastrocnemius muscle. As illustrated in the gross images of the isolated gastrocnemius muscles (Figure 4(a)), the operative side in the PNI and vehicle groups rather than FGF13 group was obviously atrophic when compared with the corresponding normal side. Moreover, quantification of relative wet weight of gastrocnemius muscle results showed that there were little muscle fiber atrophy in the FGF13 group compared with the PNI and vehicle groups (Figure 4(c)). H&E staining revealed that the average percentage of muscle fiber positive area in the FGF13 group was significantly higher than those in the PNI and vehicle groups (Figures 4(b) and 4(d)), suggesting that FGF13 holds great promising to reverse muscle atrophy in rats with sciatic nerve transection.

### 3.3. FGF13 Improves Morphological Restoration of the Regenerated Sciatic Nerve after PNI

**Figure 5:**
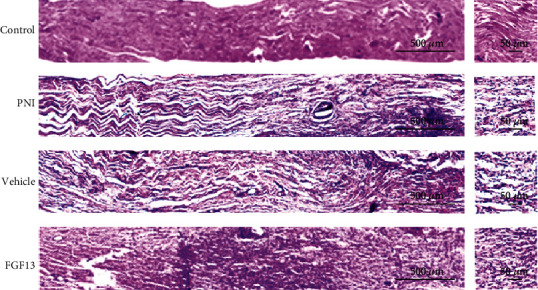
FGF13 promotes nerve regeneration after PNI. H&E staining was used to visualize the regenerated nerve fibers in the longitudinal sections of the regenerated segments in the control group, the PNI group, the vehicle group, and the FGF13 group at week 6 after the surgery. Scale bars, 500 *μ*m (a); 50 *μ*m (b).

To investigate whether overexpressing FGF13 had a protective effect on enhancing nerve regeneration, the histological changes of the regenerated axon and fibrotic scar formation in each group were observed by H&E and Masson's trichrome staining. As shown in Figure 5, the control group had uniform and dense nerve fibers with structural integrity. Following 6 weeks after transection injury, the morphological observation of regenerated nerve fibers was exhibited, scattered, and disordered, while this abnormal nerve structure was significantly improved after injection of the recombinant FGF13 lentiviral vector solution.

**Figure 6:**
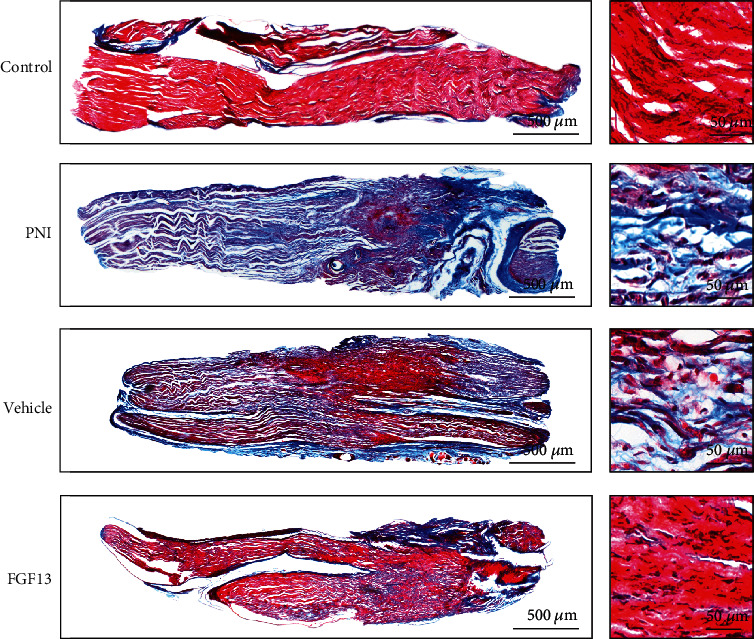
FGF13 attenuates fibrotic matrix deposition after PNI. (a) Masson's trichrome staining of the whole nerve stumps of longitudinal sections in the four groups of rats at 6 weeks after the injury. Scale bars, 500 *μ*m (a); 50 *μ*m (b).

Through Masson's trichrome staining, we could clearly observe that the amount of regenerative axons which were dyed in red showed similar pattern as H&E staining, namely, control group > FGF13 group > vehicle group > PNI group (Figure 6). However, the fibrotic scar which appeared blue showed the opposite trend, manifesting in the FGF13 group contained few fibrous formation than that of the PNI group and vehicle groups (Figure 6). All of these data suggested that FGF13 was contributed to support nerve regeneration and attenuate fibrotic matrix deposition.

### 3.4. FGF13 Enhances Remyelination following PNI

At 6 weeks after surgery, the myelin morphology at the distal portion of the transection site from four surgery groups was observed by toluidine blue staining and TEM. As shown in Figures 1(a) and 1(b), the control sciatic nerves displayed normal myelinated fibers with abundant fair arrangement and thick structure. In contrast, the PNI and vehicle groups exhibited scattered myelin sheaths with abnormal structure, manifesting in irregularity and thinning. This severe myelin abnormalities achieved greatly amelioration after receiving FGF13 lentivirus remedy. Additionally, statistical analysis of myelinated indexes, including myelin sheath counts, diameter, and thickness plus G-ratio in the FGF13 group, was significantly superior to in the PNI and vehicle groups and nearly reached to the control group (Table 1).

### 3.5. FGF13 Enhances Axonal Outgrowth following Nerve Transection

To address whether axonal regeneration after transection was enhanced by FGF13 treatment, double staining for NF-200 (green) and MBP (red) was performed. We found that the regenerated sciatic nerve in the lesion region and its distal stump without treatment showed few NF-200-positive axons and MBP-tagged myelins. Moreover, most of regenerated axons at the proximal site could not pass through the lesion region to reach the distal trunk, and the distribution of these sparse axons in the lesion core exhibited irregular (Figure 2(a)). Similarly, simple blank lentiviral vector treatment did not significantly increase the axon numbers, NF-200 and MBP immunoreactivity. In contrast, rats receiving FGF13 lentivirus were filled with NF-200 and MBP positive staining signals that extended from the proximal stump along a linear path through the whole lesion region toward the distal stump (Figure 2(a)). Additionally, analysis of axon numbers, NF-200, and MBP expression at 6 weeks postsurgery showed that the FGF13 treating group had the more axon regrowth and the higher protein expression than another two operative groups despite inferior to the sham operation group (Figures 2(c)–2(e)).

### 3.6. FGF13 Induces Microtubule Stability in SCs

**Figure 7:**
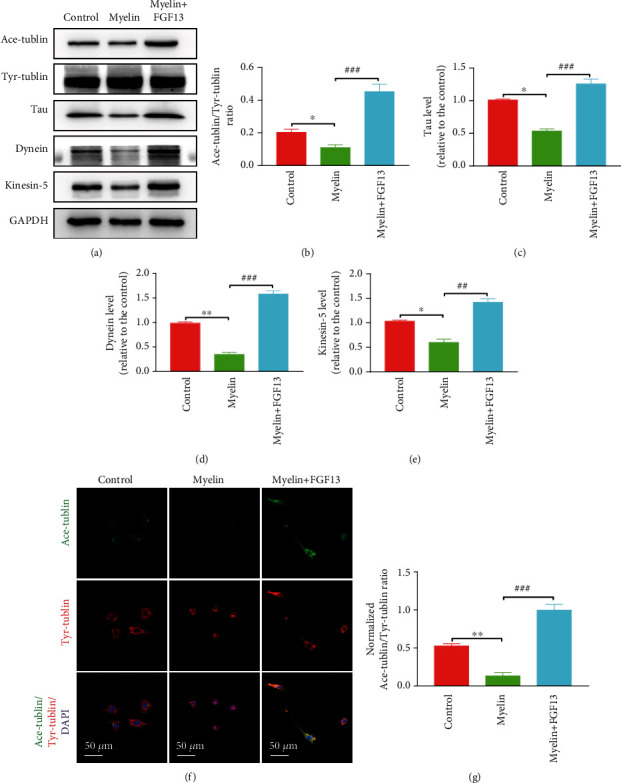
FGF13 induces microtubule stabilization in SCs. (a) Representative images of immunoblotting analysis of acetylated tubulin, tyrosinated tubulin, Tau, Kinesin-5, and Dynein in the control, myelin, and myelin+FGF13 groups. (b–e) Quantification results of the levels of the indicated proteins from (a). GAPDH was used as the loading control and for band density normalization. (f, g) Representative confocal images of Ace-tubulin and Tyr-tubulin fluorescence and quantitative results of A/T ratio in the different experimental groups. DAPI was used to label the nuclei. All these data represent the means ± SEM and sourced from three independent experiments. ^∗^*P* < 0.05 and ^∗∗^*P* < 0.01 vs. the control group; ^##^*P* < 0.01 and ^###^*P* < 0.001 vs. the myelin group.

SCs are integral components of peripheral nervous that play a pivotal role in myelin formation and axon regrowth [25]. Microtubule stability contributes to cytoskeletal remodeling to guide development and regeneration of the nervous system [26]. Microtubule stability needs to undergo frequent bouts of assembly and disassembly, which can be reflected by the relative ratio of acetylated and tyrosinated tubulins (A/T ratio) [27]. The higher its ratio, the better microtubule stabilization. Microtubule stability and dynamics are also regulated by other group of proteins, termed Tau, Dynein, and Kinesin-5. It is intuitive that tau has been proposed to regulate the assembly, growth, and bundling of microtubules in the growth cone [28]. Dynein is a complex cytoskeletal dynamic protein that drives long-range retrograde transport along microtubules [29]. Kinesin-5 is a motor protein that plays critical roles in shaping and organizing microtubules in both axons and dendrites [30]. To reveal the molecular mechanism of FGF13 promoting structural and functional recovery following PNI, we cultured SCs in myelin condition to imitate the local microenvironment of damaged sciatic nerve and detected the level of microtubule-related proteins following transfection of LV-FGF13 via immunoblotting and immunofluorescence detection. As shown in Figure 7(a), Ace-tubulin protein was expressed normal in the control group but became very low when cultured in myelin condition. After infecting with LV-FGF13, the low level of Ace-tubulin was restored to the degree that was higher than the control group. Similarly, the trend of Tau, Dynein, and Kinesin-5 expression in control, myelin, and myelin+FGF13 groups was consisted with the change of Ace-tubulin expression (Figure 7(a)). Moreover, statistical analysis also revealed that the values of Tau, Dynein, and Kinesin-5 as well as A/T ratio in the myelin+FGF13 group were the highest in all testing groups and showed statistical significance when compared with the myelin group (Figures 7(b)–7(e)). Additionally, double immunostaining for Ace-tubulin and Tyr-tubulin also showed that treatment with LV-FGF13 caused a marked increase in the A/T ratio, accompanied by cellular outgrowth with a single polarized morphology, when compared with the myelin group (Figures 7(f) and 7(g)).

### 3.7. FGF13 Reverses Myelin-Induced SC Apoptosis

**Figure 8:**
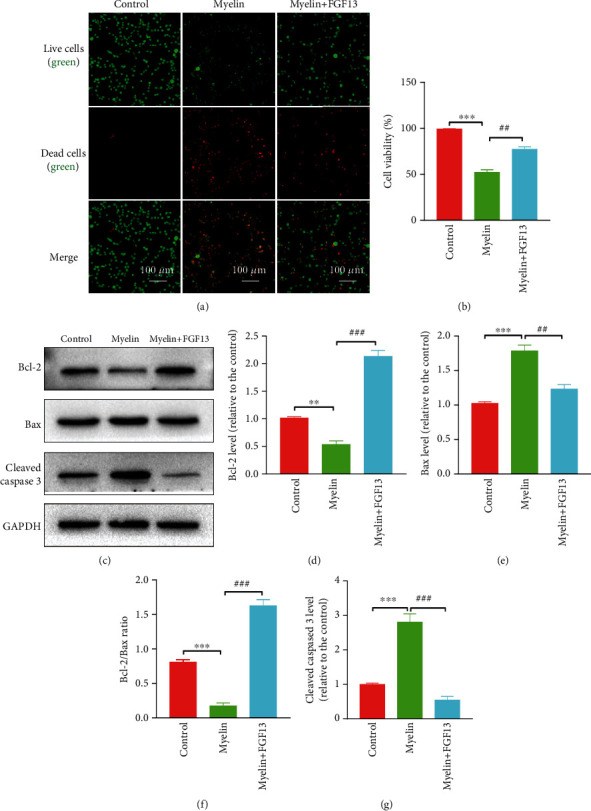
FGF13 alleviates cell death *in vitro*. (a) Live-dead staining was used to identify live (green) and dead (red) cells in the control, myelin, and myelin+FGF13 groups. (b) Quantification of cell viability from (a). (c) Representative immunoblotting images of Bcl-2, Bax, and Cleaved caspase-3 in the control, myelin, and myelin+FGF13 groups. GAPDH expression was used for normalization of protein loading. (d–g) Quantification of protein bands from (c) by densitometric analysis. The values represent the mean ± SEM and repeated in triplicate. ^∗^*P* < 0.05 and ^∗∗^*P* < 0.01 vs. the control group; ^##^*P* < 0.01 and ^###^*P* < 0.001 vs. the myelin group.

To examine whether FGF13 could alleviate SC apoptosis under damage condition, we transfected SC with lentivirus that overexpressed FGF13, followed by addition of myelin extracts. Using live/dead assay (Figures 8(a) and 8(b)), we found that there were nearly no dead cells in the normal condition, but this status became seriously terrible when SC exposed to myelin debris, manifesting in numerous cellular death or necrosis. Addition of LV-FGF13 remarkably decreased myelin-induced SC death, as evidenced by a dramatic increase in green signals (live cells) and decrease in red signals (dead cells). Similar changes were also observed for the apoptosis-related proteins in the three experimental groups (Figures 8(c)–8(g)). Specifically, compared with the normal condition, addition of myelin debris significantly upregulated the level of proapoptotic proteins, including Bax and Cleaved caspase-3, and downregulated the antiapoptotic protein Bcl-2 level, while pretreatment with LV-FGF13 effectively reversed this trend. These results suggest that LV-FGF13 is able to attenuate SC death and apoptosis at the condition of myelin-induced microenvironmental disorder.

## 4. Discussion

The results of present study support the role of FGF13 possess favorable biological property for exerting neuroprotection and neuroregeneration after transection of the sciatic nerve. Our results provide evidence that, following PNI, overexpressing FGF13 not only substantively ameliorated sensory and motor functional recovery but also remarkably promoted the morphological and pathological alterations, including axonal regrowth, myelin rehabilitation, and fibrotic scar reduction, as well as apoptotic decrease. Importantly, these benefit functions were closely associated with maintaining microtubule stabilization in SCs. Additionally, FGF13 was not participated in modulating nerve injury-induced inflammatory reaction (Figure S1 and Table. S1). In brief, these findings provide new insights into the beneficial effects of FGF13 in nerve reconstruction and functional restoration and open a possible investigation of applying FGF13 for treating PNI.

Microtubule network, contained in growth cone, guides axonal elongation and bending via interacting with microtubule-related proteins, including acetylated tubulin, tyrosinated tubulin, Tau, Kinesin-5, and Dynein [29]. During the process of cellular polarization and maturation, these proteins are gradually increased to stabilize microtubules that are contribute to cytoskeletal assembly [31]. Spatiotemporal regulation of cytoskeleton structures is beneficial for ameliorating intrinsic axon growth capacity [32, 33]. Conversely, destruction of microtubule stabilization using nocodazole impairs neuronal polarization, axon formation, and reconnection [34]. Thus, detecting the expression of microtubule-related proteins holds greatly promise for reflecting intrinsic growth capacity of damaged nerve system. In this study, we found SCs exposed to myelin debris significantly decreased the level of Tau, Kinesin-5, and Dynein expression, as well as reduced A/T ratio, but was strikingly reversed after receiving FGF13 treatment. These dates indicate FGF13-induced microtubule stabilization in SCs possibly become an intrinsic capacity for improving nerve structural and functional recovery following sciatic nerve transection injury.

After suffering from trauma and medical disorders, especially for transection of axons, adult nerve tissue regeneration is usually slow and incomplete due to the pathophysiologic disturbance-induced cell death, nerve demyelination, conduction defects, and/or muscle denervation. To achieve an effective nerve regeneration, therapeutic strategy should be possessed requirements as followings: first, stimulating microtubule protrude toward the growth cone leading edge to increase their intrinsic growth capacity [35, 36], and second, supplement of adequate biological molecules, such as GFs, in the lesion region to create a regenerative microenvironment for numerous axonal extension toward the target organ [37]. Finally, the proximal segment of axonal regrowth should be elongate at a right direction to match the corresponding injured target organs [38]. Our preliminary studies indicated that, compared with the model without treatment, FGF13 therapy shows great potential for regulating microtubule dynamic, enabling axon outgrowth and myelin regeneration, as well as promoting the locomotor recovery. To the best of our knowledge, this reason might be closely related to FGF13 as a microtubule-stabilizing protein that possess a capable for enhancing nerve intrinsic growth potential to achieve structural and functional recovery following PNI.

As an intracellular protein of the GF family, FGF13 can modulate initiation and propagation of action potentials via modulating Nav channel gating and trafficking in the developing nerve system [39]. Presently, the reasons for selection of FGF13 lentivirus for restoring severe sciatic nerve transection recovery are based on the following factors: (I) FGF13 has been regarded as a microtubule stabilizing protein that accelerates neural development and polarization and showed neuroprotective and neuroregenerative roles in promoting axonal growth and remyelination after SCI [13, 15]; (II) deficiency of FGF13 gene exhibits learning and memory decline, as well as synaptic excitatory–inhibitory imbalance [40]; and (III) in vivo transduction of wide variety of cells with lentivirus that overexpress FGF13 resulting in continuously repairing PNI over several months. Thus, we speculate that overexpression of FGF13 is a potential strategy for improving morphological and functional recovery following PNI. To confirm this speculation, the adult rats with sciatic nerve of transection lesion were injected in situ FGF13 lentivirus. At determined time point, histological and functional recovery in the lesion region was evaluated via multiple comprehensive experiments. The result of our data demonstrated that application of FGF13 significantly ameliorated locomotor outcome and axonal and myelin regeneration, as well as fibrotic deposition, indicating FGF13 as a therapeutic agent not only supported neuroregeneration in central nervous system but also improved axonal generation and plasticity in peripheral nervous system.

In summary, our data provided the first direct evidence that FGF13 had the capacity for significantly promoting axonal regrowth, remyelination, and functional reinnervation after sciatic nerve transection injury. Moreover, the underlying molecular mechanism was probably through stabilizing microtubule in SCs to enhance their intrinsic growth capacity. The findings of this exploratory study suggest that FGF13 may serve as a novel therapeutic agent for repairing PNI or other PNS injury.

## Figures and Tables

**Table 1:** Morphometric evaluations of regenerated nerves in each group.

Group	Myelin counts (/mm^2^)	Diameter of myelin sheath (*μ*m)	Thickness of myelin sheath (*μ*m)	G-ratio (axon to myelin diameter ratio)
Sham	33.950 ± 2773	5.11 ± 0.22	1.21 ± 0.09	0.63 ± 0.03
PNI	15.715 ± 1333^∗∗∗^	3.41 ± 1.26^∗∗∗^	0.56 ± 0.04^∗∗∗^	0.84 ± 0.02^∗∗∗^
Vehicle	16.557 ± 1381	2.56 ± 0.19	0.57 ± 0.07	0.82 ± 0.02
FGF13	23.293 ± 2197^&,#^	3.62 ± 0.18^&&,##^	0.83 ± 0.06^&,#^	0.68 ± 0.02^&,##^

Statistical analysis of the nerve fiber density, diameters, thickness, and G-ratios in all groups using the ImageJ software. G-ratio was calculated by dividing the diameter of the axon by fiber diameter. The data are expressed as the mean ± SEM. PNI vs. control: ^∗∗∗^*P* < 0.001; FGF13 vs. vehicle: ^&^*P* < 0.05 and ^&&^*P* < 0.01; FGF13 vs. PNI: ^#^*P* < 0.05 and ^##^*P* < 0.01.

## Data Availability

The datasets used and analyzed during the current study are available from the corresponding author on reasonable request.

## Supplementary Materials

Supplementary Materials Real-time quantitative PCR. According to the manufacturer's protocol, sciatic nerve tissues were collected and homogenized in TRIZOL (Invitrogen, California, CA, USA) to extract total RNA. Both reverse transcription and quantitative PCR (qPCR) were carried out using a two-step M-MLV Platinum SYBR Green qPCR SuperMix-UDG kit (Invitrogen, Carlsbad, CA). An Eppendorf Real plex 4 instrument (Eppendorf, Hamburg, Germany) was used to conduct real-time qPCR. The primers of target genes are listed in the Supplementary Table 1. The relative amount of mRNA was calculated by the comparative threshold cycle method with *β*-actin as control. Supplementary Figure S1: effect of LV-FGF13 on regulating inflammation response at 7d post-injury. (a) mRNA levels of the proinflammatory cytokines IL-6, IL-1*β*, and TNF-*α* in sciatic nerve tissue. (b) Gene expression of anti-inflammatory cytokines IL-4, IL-10, and IL-13 in sciatic nerve tissue from the control, PNI, vehicle, and FGF13 groups. Data are expressed as mean ± SEM for three independent experiments. ^∗∗∗^Statistically significant difference (*P* < 0.001) versus the control group. No statistical significance (n.s) was observed between PNI and FGF13 groups, plus vehicle, and FGF13 groups. Supplementary Table 1: primer sequences for real-time qPCR.
