# Supplementary material for: Fibroblast Growth Factor 13 Facilitates Peripheral Nerve Regeneration through Maintaining Microtubule Stability
Source: Oxid Med Cell Longev. 2021 Aug 20;2021:5481228. doi: 10.1155/2021/5481228 (PMC8397546; doi:10.1155/2021/5481228)
Supplement: Supplementary Materials — Real-time quantitative PCR. According to the manufacturer's protocol, sciatic nerve tissues were collected and homogenized in TRIZOL (Invitrogen, California, CA, USA) to extract total RNA. Both reverse transcription and quantitative PCR (qPCR) were carried out using a two-step M-MLV Platinum SYBR Green qPCR SuperMix-UDG kit (Invitrogen, Carlsbad, CA). An Eppendorf Real plex 4 instrument (Eppendorf, Hamburg, Germany) was used to conduct real-time qPCR. The primers of target genes are listed in the Supplementary Table 1. The relative amount of mRNA was calculated by the comparative threshold cycle method with β-actin as control. Supplementary Figure S1: effect of LV-FGF13 on regulating inflammation response at 7d post-injury. (a) mRNA levels of the proinflammatory cytokines IL-6, IL-1β, and TNF-α in sciatic nerve tissue. (b) Gene expression of anti-inflammatory cytokines IL-4, IL-10, and IL-13 in sciatic nerve tissue from the control, PNI, vehicle, and FGF13 groups. Data are expressed as mean ± SEM for three independent experiments. ∗∗∗Statistically significant difference (P < 0.001) versus the control group. No statistical significance (n.s) was observed between PNI and FGF13 groups, plus vehicle, and FGF13 groups. Supplementary Table 1: primer sequences for real-time qPCR. [file 5481228.f1.docx]

**Fibroblast growth factor 13 facilitates peripheral nerve regeneration through maintaining microtubule stability**

Rui Li ^a,b †*^, Xuetao Tao ^c †^, Minghong Huang ^a †^, Yan Peng ^d^ , Jiahong Liang ^e^, Yanqing Wu ^f *^, Yongsheng Jiang ^a *^

^a^ The Affiliated Xiangshan Hospital of Wenzhou Medial University, No. 291 Donggu Road, Xiangshan County, Zhejiang 315000, China

^b^ PCFM Lab, GD HPPC Lab, School of Chemistry, Sun Yat-sen University, Guangzhou 510275, China.

^c^ The Second Affiliated Hospital, Zhejiang University School of Medicine, Hangzhou, Zhejiang, 310009, China

^d^ Hangzhou Institute for Food and Drug control, Hangzhou, Zhejiang, 310014, China

^e^ Betta Pharmaceuticals Co., Ltd. Hangzhou, Zhejiang 310000, China

^f^ The Institute of Life Sciences, Engineering Laboratory of Zhejiang Province for Pharmaceutical Development of Growth Factors, Biomedical Collaborative Innovation Center of Wenzhou, Wenzhou University, Wenzhou, Zhejiang, China 325035

**Correspondence should be addressed to**

Rui Li; E-mail: xiaoerrui1989@163.com; Yanqing Wu; E-mail: yqwu220946@yeah.net and Yongsheng Jiang; E-mail: shenren34127@163.com

^†^ These authors contribute equally to this work.

**Supplementary methods**

**Real-time quantitative PCR** According to the manufacturer's protocol, sciatic nerve tissues were collected and homogenized in TRIZOL (Invitrogen, California, CA, USA) to extract total RNA. Both reverse transcription and quantitative PCR (qPCR) were carried out using a two-step M-MLV Platinum SYBR Green qPCR SuperMix-UDG kit (Invitrogen, Carlsbad, CA). An Eppendorf Real plex 4 instrument (Eppendorf, Hamburg, Germany) was used to conduct real-time qPCR. The primers of target genes are listed in the Supplementary Table 1. The relative amount of mRNA was calculated by the comparative threshold cycle method with β-actin.as control.


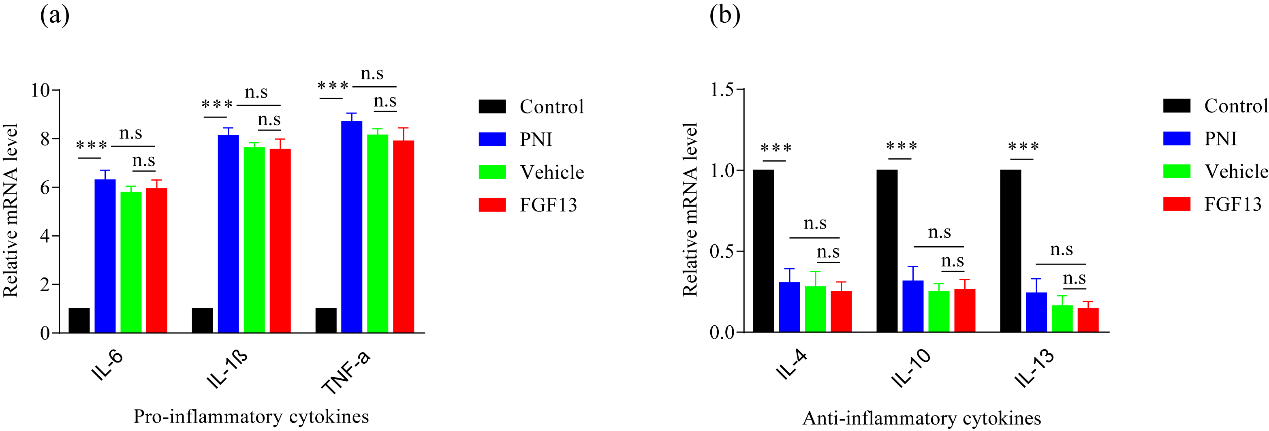


**Supplementary Figure S1.** Effect of LV-FGF13 on regulating inflammation response at 7d post-injury. (a) mRNA levels of the pro-inflammatory cytokines IL-6, IL-1β and TNF-α in sciatic nerve tissue. (b) Gene expression of anti-inflammatory cytokines IL-4, IL-10 and IL-13 in sciatic nerve tissue from the control, PNI, vehicle and FGF13 groups. Data are expressed as mean ± SEM for three independent experiments. ^***^Statistically significant difference (*P* < 0.001) versus the control group. No statistical significance (n.s) was observed between PNI and FGF13 groups, plus vehicle and FGF13 groups.

Supplementary Table 1: Primer sequences for real-time qPCR

| **Gene** | **Species** | **Forward primer (5’-3’)** | **Reverse primer (5’-3’)** |
| --- | --- | --- | --- |
| IL−6 | Rat | GAGTTGTGCAATGGCAATTC | ACTCCAGAAGACCAGAGCAG |
| IL-1β | Rat | GGGCCTCAAGGGGAAGAATC | ATGTCCCGACCATTGCTGTT |
| TNF-α | Rat | AGGAGGAATTTGGCCAGGTG | GCTCACGAGGAGGCTAATCC |
| IL-4 | Rat | GAGGAGAGGGCGGACATT | ACTCTTCATTCAGGCCCTTG |
| IL-10 | Rat | GAGGAGAAGATGCCGGTAG | TCAGAGAGGGAGCTAAGTG |
| IL-13 | Rat | CCTAAAACAACCTCAGCCCGT | TTCCGGATCCAGGAGAGACTT |
| β-actin | Rat | AAGTCCCTCACCCTCCCAAAAG | AAGCAATGCTGTCACCTTCCC |
